# Editorial: Biophotoelectrochemistry for the nexus of energy and environment

**DOI:** 10.3389/fmicb.2022.1072083

**Published:** 2022-11-01

**Authors:** Jie Ye, Yong Yuan, Yifeng Zhang, Wulin Yang, Xiang Gao

**Affiliations:** ^1^Fujian Provincial Key Laboratory of Soil Environmental Health and Regulation, College of Resources and Environment, Fujian Agriculture and Forestry University, Fuzhou, China; ^2^Guangdong Key Laboratory of Environmental Catalysis and Health Risk Control, School of Environmental Science and Engineering, Institute of Environmental Health and Pollution Control, Guangdong University of Technology, Guangzhou, China; ^3^Department of Environmental Engineering, Technical University of Denmark, Lyngby, Denmark; ^4^College of Environmental Science and Engineering, Peking University, Beijing, China; ^5^CAS Key Laboratory of Quantitative Engineering Biology, Shenzhen Institute of Synthetic Biology, Shenzhen Institute of Advanced Technology, Chinese Academy of Sciences (CAS), Shenzhen, China

**Keywords:** semiconductor synthetic biology, electron transfer, photo-biocatalysis, semi-artificial photosynthesis, biohybrids

Electron exchange is a well-established driving force in microbial ecology. Diverse electroactive microorganisms including exoelectrogens and electrotrophs are proven to interact with various microbial species, minerals or soluble electron acceptors/donors *via* extracellular electron transfer (EET); that is, electromicrobiology (Shi et al., 2016; Lovley and Holmes, 2022). Such a process effectively promotes the construction of a complex social network of microorganisms in various electromicrobiomes. In recent years, a new picture has emerged, emphasizing that photoelectrons from photosensitizers, such as semiconductors, photosensitizing proteins and photoautotrophs, can participate in EET and then influence the physiological metabolism activity of electroactive microorganisms or biofilms (Kornienko et al., 2018). This brings an emerging frontier discipline, named biophotoelectrochemistry (BPEC) (Ye et al., 2021). The relevant research results may provide insight supporting vital photon-induced multiple biogeochemical redox processes (e.g., the interaction between microbe-rock/mineral, microbe-microbe), also a new metaphorical branch of microbial evolution.

This Research Topic aimed to explore recent developments in BPEC field with a focus on (1) Novel BPEC systems or material biohybrids for solar-driven biocatalysis *via* advanced technologies; (2) extracellular electron transfer mechanisms of electroactive microorganisms such as direct interspecies electron transfer (DIET); (3) new applications of BPEC or new systems that are derived from BPEC for environmental engineering application.

Photosensitizer is an important component of BPEC system. However, the commonly used photosensitizers such as semiconductor quantum dots are limited by the toxicity of involved heavy metal elements. To address this challenge, the first study by Hu et al. constructed a biotic-abiotic hybrid system by integrating metal-free black phosphorus/carbon nitride (BPCN_x_) with *Methanosarcina barkeri* (*M. b*) for methanogenesis. The results showed that a superior CH_4_ yield of 1,087.45 ± 29.14 ${\text{μmol·g}}_{\text{cat}}^{-1}$ was achieved with *M. b*-BPCN_x_ after three-cycle operation under light illumination. This is because that BPCN_x_ in hybrid system not only had an excellent separation efficiency of the electrons-hole pairs, but also effectively maintained the activity of methanogens. These results open a new avenue to develop an environmentally-friendly and cost-effective hybrid system for the production of renewable energy.

DIET is an important mechanism for microbial species to exchange electrons cooperatively during syntrophic metabolism. The addition of conductive materials can improve the DIET efficiency during anaerobic digestion. Chen et al. comprehensively summarized the underlying mechanisms of how DIET mediated by conductive materials influences the lag phase, methane production, and system stability of anaerobic digestion. Furthermore, current challenges such as the unclear biological mechanisms, influences of non-DIET mechanisms and problems in practical application are discussed in detail. Finally, the future research directions for practical application of DIET are outlined.

In addition, it is generally accepted that DIET is mainly mediated by electrically conductive pili and outer surface c-type cytochromes (c-Cyts). However, exopolysaccharides are the ubiquitous and abundant extracellular matrices on the surface of microorganisms, and their effects on DIET is still unclear. Zhuang et al. constructed a co-culture of exopolysaccharides-deficient *Geobacter sulfurreducens* with *Geobacter metallireducens* to explore the role of exopolysaccharides on DIET. Results revealed that the deficiency of exopolysaccharides extended the metabolic period of the co-culture by 44.4%, and changed the proportions of each species in the co-culture. The exopolysaccharides-deficient co-culture failed to form large, tight spherical aggregates, and the expression of c-Cyts and pili was decreased. These findings demonstrate that non-conductive exopolysaccharides are an important component of DIET aggregates and an extracellular matrix for DIET-required c-Cyts.

Another study by Wen et al. tied to evaluate the underlying electron transfer mechanisms during the ectopic fermentation system (EFS). The results showed a noticeable transformation of protein-like substances into humus-like substances, along with the increased persistently electron–accepting capacity. Particularly, the contents of phenols that promoted electron transfer continued to increase from 2.80 to 6.00%, which could be used as a maturity indicator for EFS. This study provides insights into understanding the humification mechanisms and implementing regulatory strategies.

As guest editors of this Research Topic, we would like to thank all the authors and reviewers for their valuable contributions to this special issue. We expect that the readers find this topic issue of interest.

## Author contributions

All authors listed have made a substantial, direct, and intellectual contribution to the work and approved it for publication.

## Funding

This work was supported by the National Natural Science Foundation of China (42177206) and the Natural Science Foundation of Fujian Province, China (2020J06017).

## Conflict of interest

The authors declare that the research was conducted in the absence of any commercial or financial relationships that could be construed as a potential conflict of interest.

## Publisher's note

All claims expressed in this article are solely those of the authors and do not necessarily represent those of their affiliated organizations, or those of the publisher, the editors and the reviewers. Any product that may be evaluated in this article, or claim that may be made by its manufacturer, is not guaranteed or endorsed by the publisher.
